# Strongly Magnetic Iron Nanoparticles Improve the Diagnosis of Small Tumours in the Reticuloendothelial System by Magnetic Resonance Imaging

**DOI:** 10.1371/journal.pone.0056572

**Published:** 2013-02-20

**Authors:** Peter M. Ferguson, Kirk W. Feindel, Angela Slocombe, Matthew MacKay, Trudy Wignall, Brett Delahunt, Richard D. Tilley, Ian F. Hermans

**Affiliations:** 1 Malaghan Institute of Medical Research and School of Biological Sciences, Victoria University of Wellington, Wellington, New Zealand; 2 Department of Pathology and Molecular Medicine, Wellington School of Medicine and Health Sciences, University of Otago, Wellington, New Zealand; 3 National Research Council Institute for Biodiagnostics (Atlantic), Halifax, Nova Scotia, Canada; 4 Department of Radiology, Wellington Hospital, Wellington, New Zealand; 5 School of Chemical and Physical Sciences, The MacDiarmid Institute for Advanced Materials and Nanotechnology, Victoria University of Wellington, Wellington, New Zealand; Centro di Riferimento Oncologico, IRCCS National Cancer Institute, Italy

## Abstract

Despite advances in non-invasive medical imaging, accurate nodal staging of malignancy continues to rely on surgery. Superparamagnetic iron oxide nanoparticles (IONP) with lymphotropic qualities have shown some promise as contrast agents for MRI of the lymph nodes, but recent large-scale studies failed to show consistent detection of tumours below 5 mm. Herein we compare imaging of splenic and lymph node tissue using iron/iron oxide core/shell nanoparticles (Fe NP) that have superior magnetic qualities to IONP, to determine whether improved negative contrast in *T_2_*-weighted MRI can enhance the diagnosis of small tumours in the reticuloendothelial system. To provide an *in vivo* pre-clinical model of human lymph node micrometastases, breast cancer cells were injected into the spleens of mice, providing localised areas of tumour growth. MR images of groups of tumour-bearing and sham-treated animals were generated using a 1.5 T imaging system and analysed by two independent, blinded radiologists. Fe NP improved the sensitivity and specificity of MRI when compared to IONP, enabling accurate detection of tumours as small as 1–3 mm. The use of Fe NP as contrast agents have the potential to improve the diagnostic accuracy of MRI in cancer patients, leading to more rapid and effective treatment.

## Introduction

Early and accurate detection of metastases in the lymph nodes has been shown to improve patient outcome in a number of different malignancies [1]–[3]. While cross-sectional MRI of lymph nodes has been relatively insensitive in detecting nodal metastases [4], some promise has been shown in developing lymph-node specific MRI contrast agents to improve image interpretation [5]–[7]. Such contrast-enhanced MRI relies on the lymphotropic properties of superparamagnetic nanoparticles (NPs), which are internalized by phagocytes of the reticuloendothelial system (RES), causing local changes in magnetic properties [8], [9]. *T_2_*-weighted sequences are especially sensitive to the magnetic field changes induced by the intracellular NPs; in healthy liver, spleen and lymph node tissue this is demonstrated as a decrease in signal intensity as phagocytic cells acquire the NPs, whereas malignant tissue generally fails to acquire NPs and appears bright relative to the surrounding tissue.

The use of IONP for contrast-enhanced MRI has been shown to improve detection of tumours greater than 5 mm in diameter in the lymph nodes when compared to unenhanced MRI or computed tomography [8], [10], and is also more effective when the tumours cause lymph node enlargement [11]. However, the sensitivity of detecting tumours decreases substantially if the size is less than 5 mm [8]. Recent phase III studies into the use of IONP for non-invasive nodal evaluation with MRI in patients with a variety of malignancies highlighted several shortcomings, including inability to demonstrate a consistent and significant benefit in sensitivity and specificity, and significant inter-observer variability (9).

We recently developed a novel synthesis for iron/iron-oxide core/shell NPs (Fe NP) with superior magnetic properties that translate into greater *T_2_* relaxivity in MRI with a similarly low cytotoxicity when compared to IONP of similar size (16 nm) and coated with the same molecule, dimercaptosuccinic acid [12]–[14]. We hypothesised that due to the improved *T_2_*-relaxivity of Fe NP, the threshold of detection of tumours in RES tissue can be reduced to below 5 mm in size without sacrificing diagnostic accuracy. This was tested in a murine model in which breast cancer cells were engrafted into the spleen as a model of a micrometastatic lesion within RES tissue, similar in size to a small metastasis within a human lymph node. The results presented show improved diagnosis using Fe NP and demonstrate progress toward removing the need for dedicated radiologists specifically trained in contrast-enhanced MRI to achieve high accuracy.

## Methods

### Ethical Statement

Experiments were conducted in accordance with the Animal Ethics Policy 2008R7M, approved by the Animal Ethics Committee of Victoria University of Wellington. All surgery and MRI scans were performed under ketamine/xylazine anaesthesia, multimodal postoperative analgesia was administered and all efforts were made to minimize suffering.

### Manufacture of Nanoparticles for Use as Contrast Agents

Manufacture of Fe NP was as reported [13]. Briefly, the iron precursor [Fe(C_5_H_5_)(C_6_H_7_)] was combined with oleylamine and mesitylene (both Sigma-Aldrich, St Louis, MO, USA) in a closed reaction vessel under 100 kPa H_2_, heated to 130°C for 2 days, and then cooled to room temperature before being opened to air. Ligand exchange to replace oleylamine with meso-2,3-dimercaptosuccinic acid (DMSA, 98% Sigma-Aldrich) was used to improve water solubility of the NPs [15], which were dispersed in water or sterile PBS, sterile-filtered, and stored at 4°C until use. For comparison as contrast agents, IONP were synthesized following a commonly adopted method [16], and DMSA coating applied using the same ligand exchange procedure.

### Mice

Breeding pairs of BALB/c mice were originally sourced from the Australian Research Council, Canberra, ACT, Australia, and then bred and maintained in the Biomedical Research Unit of the Malaghan Institute of Medical Research.

### Implantation of 4T1 Breast Cancer Cells

The mouse breast cancer line, 4T1 was obtained from American Type Culture Collection (Manassas, VA, USA) and were maintained in culture for no more than an additional 20 passages from the original vial purchased. The 4T1 cells were cultured in DMEM (Invitrogen, Auckland, NZ) supplemented with 20% FBS (Sigma-Aldrich), 2 mM glutamax, 100 U/mL penicillin and 100 µg/mL streptomycin (all Invitrogen). For implantation into the spleen, 6–8 week-old BALB/c mice were anesthetized by intraperitoneal injection of a ketamine (100 mg/kg; Phoenix Pharm, Auckland) and xylazine (10 mg/kg, Phoenix Pharm), and then the spleen was exposed and injected with 1×10^6^ 4T1 cells in 50 µl of sterile PBS. The peritoneum and skin were closed with 5–0 Vicryl Rapide sutures (Johnson & Johnson Medical, Auckland, NZ) and the mice provided analgesia with one subcutaneous dose (0.1 mg/kg) of buprenorphine (Renckitt Benckiser Pharmaceuticals, Richmond, VA, USA) and two subcutaneous doses (5 mg/kg) of carprofen (Norbrook Laboratories, Auckland, NZ), 12 h apart. Experiments were performed with a total of 60 mice, with 30 receiving an intrasplenic injection of 4T1 tumour cells, and 30 sham-treated with PBS only.

### Contrast Agent Injection

Three days after intrasplenic injection the mice were randomly assigned to three groups to receive Fe NP, IONP, or Phosphate-buffered saline (PBS) only (control) respectively. All injections were by intravenous injection via the tail vein, with the contrast agents administered as a PBS dispersion (1% Fe) at a dose of 8 mg Fe/kg.

### Magnetic Resonance Imaging

Eight hours after administration of contrast agents, the mice were anesthetized by an intraperitoneal injection as above, and lacrilube (Allergan, Irvine, CA) applied to the cornea to prevent desiccation. MRI was performed using a clinical Achieva 1.5 T MR scanner (Philips Healthcare Systems, Andover, MA, USA), equipped with a wrist solenoid coil. Two mice were positioned in the centre of the wrist coil and the same protocols were used for all images. *T_2_*-weighted spin-echo images were acquired with the following parameters: TE = 54 ms, TR = 2000 ms, field-of-view = 80 mm (right-left) ×80 mm (caudal-rostral) in coronal scans or 20 mm (anterior-posterior) in axial scans, pixel size = 300 µm×300 µm, thickness = 1 mm, number of echoes = 8, 3 averages, total experiment time = 4 min 24 s. Both an axial and a coronal series of images were obtained.

### Image Analysis

**Figure 1:**
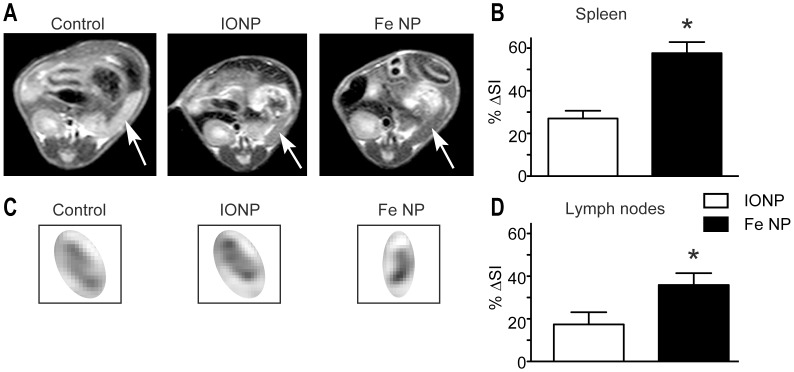
MRI contrast in the spleen and inguinal lymph nodes following administration of Fe NP or IONP. (A) Example images showing the change of signal intensity (SI) in the spleen (arrows), and (C) inguinal lymph nodes 8 h after receiving intravenous PBS (control, left), IONP (middle) or Fe NP (right). (B) The SI in the spleens and (D) lymph nodes were compared those of control mice and plotted as %ΔSI signal as follows: (SI of control - SI of subject)/(SI of control). (*P<0.05 by Mann-Whitney rank-sum test).

Images were analysed with ImageJ (National Institutes of Health, Bethesda, MD) by manually selecting regions of interest (ROIs) corresponding to the paravertebral muscle, inguinal lymph nodes, spleen or tumour and integrating the signal intensity (SI) of each ROI. For the data presented in Figure 1, where comparison of SI is made between mice, the SI of the ROI was first normalised with reference to the paravertebral skeletal muscle in the same image, as muscle is known to have minimal uptake of IONP [17].

### Histology

After MR imaging, and while still anesthetised, the mice were euthanized by cervical dislocation and their tissues (liver, spleen or tumours) were removed and to confirm presence of tumour tissue. The tissues were embedded in paraffin, sectioned at 5 µm thickness and stained with hematoxylin and eosin (H&E) stain or Prussian Blue (Perl’s) with Saffranin-O counterstain. Slides were examined under an Olympus IX51 optical microscope (Olympus, Auckland, NZ), with digital images recorded.

### Radiologist Scoring of Tumours

MR image analysis was performed by two independent, blinded radiologists both of whom had extensive experience reading clinical MRI scans but had not previously interpreted images with NP-based contrast agents. The spleens were scored on a numerical scale from one to five according to the probability of an intrasplenic tumour being present (Table 1). Both radiologists had the entire set of mouse images at one time to view on their standard clinical viewers (Carestream Health, Rochester, NY, USA). The mice were randomly assigned into pairs to be scanned within the wrist coil. The reviewers were able to look through the entire set of images before committing to a score. To address intra-observer variability, radiologist 1 was given training with a member of the research team using a random selection of images from the same image set over two thirty minute sessions. One week following training, radiologist 1 was asked to go through and score the image set independently for a second time.

**Table 1:** Numerical scoring scale and associated descriptive criteria used in reviewing MR images.

Score	Description
1	Confident that no tumour is present
2	Probably no tumour present
3	Equivocal
4	Probably a tumour present
5	Confident that a tumour is present

### Statistics

Results were plotted with error bars representing SEM. The non-parametric Mann Whitney test was used for comparison of unpaired data. All statistical analyses were done with Prism 5.0 software (GraphPad Software, Inc., La Jolla, CA), with *P*-values of <0.05 considered significant.

## Results and Discussion

### Fe NP Improve Contrast-enhancement

The spleen in 6 week old BALB/c mice has a diameter of 2–10 mm depending on the axis and orientation, which is of similar size range to the diameter of human lymph nodes when not enlarged (3–10 mm) [18]. The mouse spleen, like human lymph nodes, is also rich in phagocytic cells capable of sequestering NPs, and is therefore a useful model to examine MRI imaging of lymphoid tissue using lymphotropic NPs for contrast enhancement. Initial studies were conducted to examine *T_2_*-weighted scans of both spleen and lymph nodes after BALB/c mice were administered Fe NP, or IONP intravenously. Significantly greater decreases in signal intensity were observed in these tissues with Fe NP (Fig. 1), suggesting that Fe NP could provide superior image contrast to detect tumours in both types of RES tissue. Previous work by our group has shown that Fe NP produce more than twice the reduction in *T_2_*-weighted signal in the liver compared with IONP [12].

**Figure 2:**
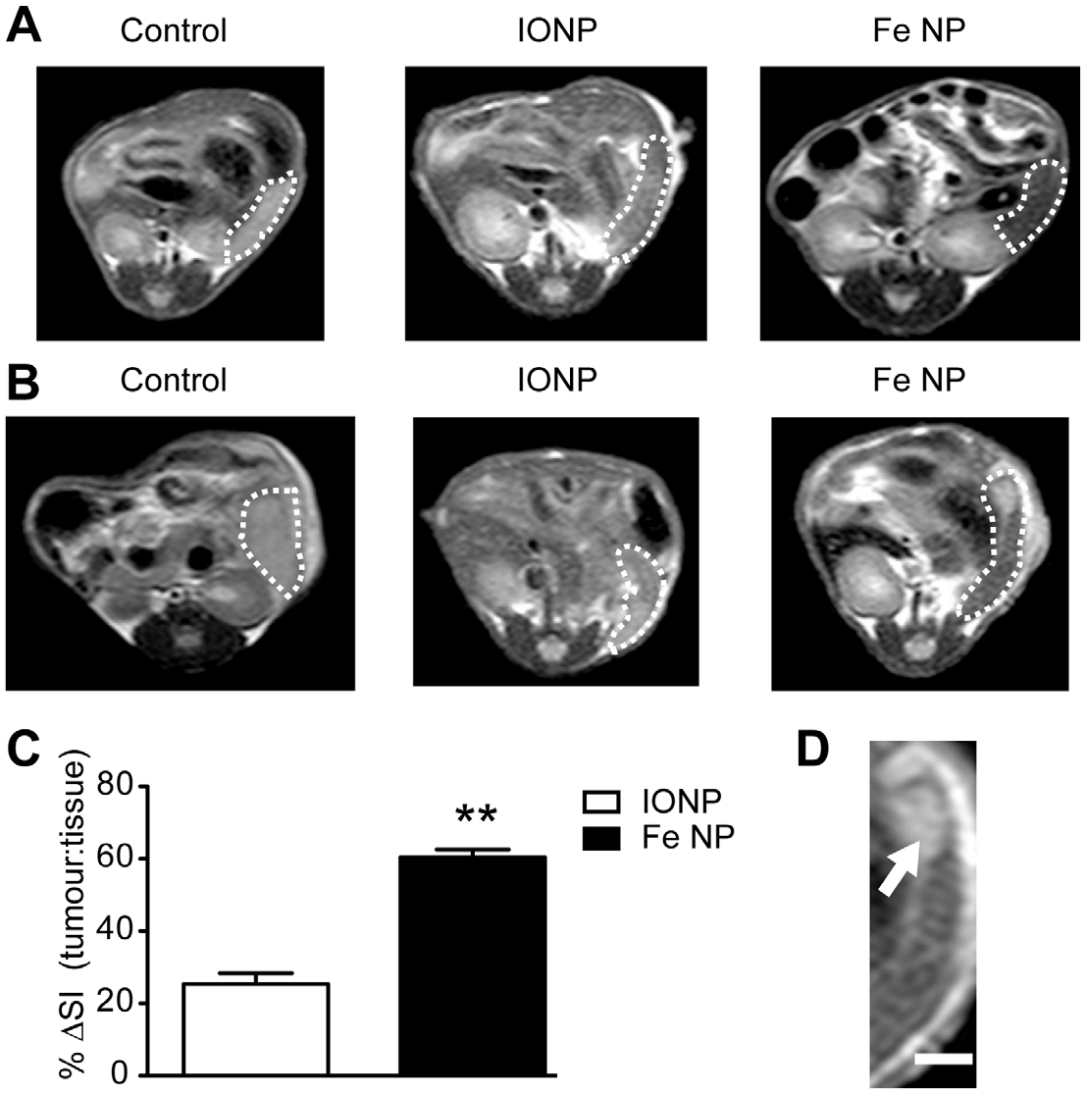
MRI contrast of small intrasplenic tumours following administration of Fe NP or IONP. (A) Six week old Balb/c mice received intrasplenic injections of PBS only, or (B), 1×10^6^ 4T1 breast cancer cells and were imaged by MR 72 h later. An intravenous dose of PBS (Control, left column), IONP (middle column) or Fe NP (right column) was administered 8 h before imaging. (C) The signal intensity (SI) in the tumours was compared within the same mouse to that of the spleen and plotted as %ΔSI signal as follows: (SI of tumour - SI of spleen)/(SI of tumour). (D) An enlarged image (scale bar = 2 mm) shows the clear distinction between splenic parenchyma and tumour (arrow) in a representative mouse that received Fe NP. (**P<0.01 by Mann-Whitney rank-sum test).

**Figure 3:**
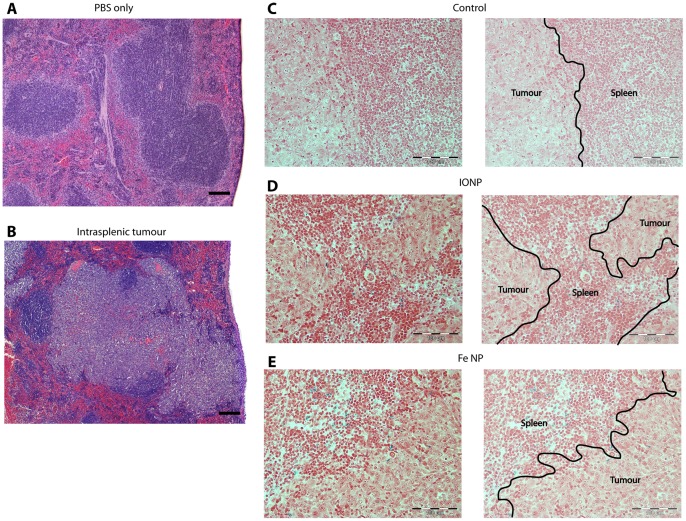
Histology of intrasplenic tumours. (A) Sections show mouse spleens 72 h after receiving intrasplenic injections of PBS only, or (B), 1×10^6^ 4T1 breast cancer cells. Following intrasplenic injections of tumour cells, mice also received intravenous injections of (C) PBS, (D) IONP or (E) Fe NP. The spleens were removed, fixed and sectioned for light microscopy. An H&E stain was applied in (A) and (B) to show the tumour architecture and a Perl stain was applied in (C-E) which demonstrates the presence of Fe (blue) predominantly in the splenic tissue, and not in the tumours, of mice that received IONP or Fe NP. (scale bar = 100 µm).

To provide an in vivo model to image RES tumours, 4T1 breast tumour cells were engrafted into the spleens of BALB/c mice 3 days before MRI was conducted. Analysis of scans revealed differences in signal intensity between tumour and normal splenic tissue (parenchyma) in animals administered either contrast agent (Fig. 2). While these differences were assumed to reflect preferential uptake of NPs by phagocytic cells within the parenchyma, accumulation of NPs at sites of tumour necrosis could be a confounding factor. The spleens were therefore removed and subject to histological analysis with Perl’s iron stain to examine NP distribution. There was a clear difference between the large, confluent tumour cells and the splenic parenchyma, with positive staining for Fe throughout the parenchyma and at the parenchyma/tumour boundary in animals administered either contrast agent, but no Fe was seen in any of the tumours (Fig. 3). No positive staining for Fe could be observed in animals that had not been administered NPs. Importantly, MRI images generated with Fe NP produced a more pronounced distinction between tumour and normal tissue than IONP (Fig. 2), with the difference between the tumour-to-tissue *T_2_* contrast ratio being 60% for scans enhanced with Fe NP versus 25% for IONP (Fig. 2C).

### Fe NP Increase Sensitivity, Specificity, and Accuracy

To assess whether the improved contrast provided by Fe NP could routinely improve the detection of small tumours in RES tissue, groups of 10 tumour-bearing and 10 sham-treated animals were subject to MRI with or without the different contrast agents, and then the scans generated were independently analysed by two blinded radiologists. The tumours were 1–3 mm in diameter, a size on the limits of detection by any current imaging protocols, and MRI was performed using a standard clinical 1.5 T device. The radiologists scored the scans on a numerical scale reflecting the likelihood of an intrasplenic tumour being present (Table 1).

**Figure 4:**
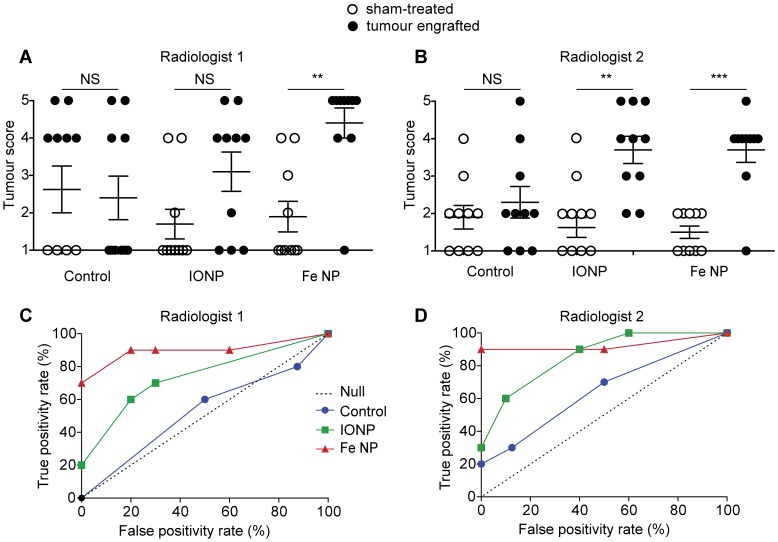
Receiver operator characteristic (ROC) curve of in-vivo tumour detection. MRI scanning was performed 72 h following tumour inoculation, with contrast administered 8 h before the scan. The scans were scored by blinded radiologists (A,C - radiologist 1; B,D - radiologist 2) according to the scale in Table 1. The results are presented as scatter plots (A, B) and ROC curves (C, D) after analysis by a non-blinded investigator. (NS *P*>0.05; **P<0.01, ***P<0.001 by Mann-Whitney rank sum test).

Interpretation of un-enhanced scans proved difficult, with both radiologists failing to accurately score the spleens, reflecting an inability to easily distinguish tumours from the splenic parenchyma. Administration of IONP improved the diagnostic accuracy of only one of the radiologists. However, administration of Fe NP significantly improved the diagnostic accuracy of both radiologists (Fig. 4A). Receiver-operator characteristic (ROC) curves were generated for each radiologist, providing a graphical plot of the true positive rate versus false positive rate (Fig. 4B).

A notable feature of both curves was a trend towards improved accuracy when Fe NP were used, highlighted by a low rate of false positives. While radiologist 2 did detect 100% of tumours when IONP were used, this was accompanied by a high false positive rate of 40%. We note that previous research has shown a large variability in specificity with IONP, which was cited as a reason for the European Medicine Authority’s decision to reject the application of IONP for use in assisting diagnosis of lymph node metastases [9].

Overall, this data provides strong evidence that the improved contrast enhancement provided by Fe NP can improve detection of small tumours in the RES by MRI at 1.5 T.

### Fe NP Decrease Inter-observer Variability and Provide Accuracy without Training

**Figure 5:**
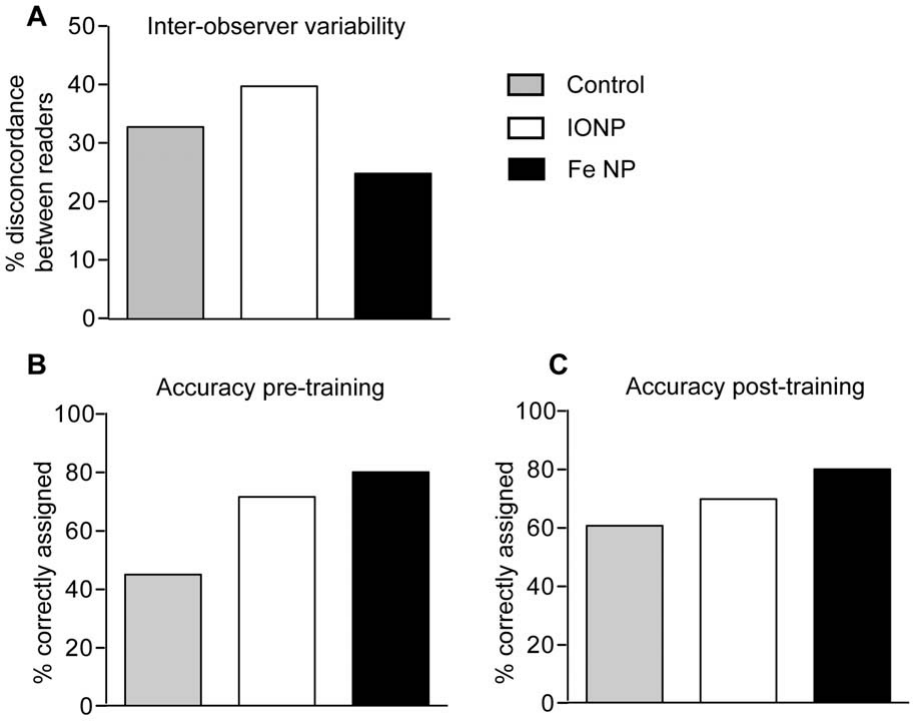
Inter- and intra-observer variability. (A) The scoring of each mouse was compared between the two radiologists. The variation between the scorings was plotted for each type of contrast agent with the two scores for each mouse defined as variant if they differed by more than one point. After an initial scoring (B), radiologist 1 received training then rescored all the mice (C), with a value of 1–2 considered correct if no tumour was present and 4–5 correct if a tumour was present.

Previous research with IONP has shown inter-observer variation in accuracy, although this could be improved through specialized training [19], [20]. The radiologists in this study had no prior experience reading contrast-enhanced MR images obtained using *T_2_* contrast agents. It was therefore notable that when scores provided by each radiologist for each mouse were compared (and defined as variant if they differed by more than one point), the inter-observer variability was lowest for scans enhanced with Fe NP (Fig. 5A).

To address whether training had any impact on scoring, one of the radiologists reviewed a random sample of images with the research team, and was then asked to re-read all of the scans one week later (Fig. 5B). This training substantially improved the scoring of un-enhanced images, indicating a better appreciation of the appearance of normal spleen in mice. While there was little change in the accuracy of scoring images enhanced with IONP or Fe NP, the accuracy of scoring was still greatest when Fe NP were used. Taken together with the greater consistency in scoring between the two observers when Fe NP were used, these results indicate that the contrast enhancement achieved with Fe NP is sufficiently distinct to facilitate consistent scoring, even without specialized training of the radiologist. Such robust reliability is ideal for widespread clinical diagnostic environments, where significant variation in expertise and experience may exist.

### Validation of MRI Contrast in Preclinical Model

The resolution of 0.3 mm×0.3 mm used in this study is higher than that used in most diagnostic MRI at the time of writing [21]–[23]. We do not perceive this to be a significant obstacle to translating these results in a clinical trial as advances in resolution, using phased-coil arrays for example, can already achieve voxels with submillimeter resolution in clinical imaging at 1.5 T [24].

To determine if the MRI contrast observed in this preclinical study provides results comparable with previous studies on humans, we consider our results with those from a recent meta-analysis of 34 studies that used IONP to diagnose lymph node tumours in humans [5]. The present study provided two similar ROC curves, with average areas under the curve of 0.90, 0.70 and 0.30 for Fe NP, IONP, and unenhanced control, respectively. Using a similar measure, the meta-analysis reported a superior pooled sensitivity of 0.91 with IONP, and 0.39 with unenhanced scans. The decreased sensitivity in our study for both IONP and the unenhanced scans is anticipated, as the smaller size of the tumours makes for inherently more challenging diagnostics. In addition, the studies compared in the meta-analysis were not all blinded and many of the investigators scoring the tumours had prior research experience with IONP, which may improve accuracy. Taken in this light, the average sensitivity of 0.90 achieved with Fe NP is impressive, and suggests that if translated from this preclinical model to the clinic, should lead to more accurate diagnosis of smaller tumours.

### Conclusions

This study demonstrates that the Fe NP described here increase sensitivity, specificity, and accuracy of tumour detection relative to unenhanced MRI, or contrast enhancement with IONP. Analysis by two independent, blinded radiologists provides robust evidence that the improved contrast enhancement with Fe NP facilitates detection of tumours as small as 1 mm, and reduces inter-observer variability. Although this study employed a splenic tumour model, Fe NP have also demonstrated a similar magnitude of *T_2_*-weighted signal contrast over IONP in the liver and lymph nodes. With these advantages we envision improved diagnostic accuracy of metastases in human RES tissue, enabling more effective staging and treatment of cancer.
